# Angiopoietin-like protein 4 (ANGPTL4) is related to gestational weight gain in pregnant women with obesity

**DOI:** 10.1038/s41598-018-29731-w

**Published:** 2018-08-20

**Authors:** Henar Ortega-Senovilla, Mireille N. M. van Poppel, Gernot Desoye, Emilio Herrera

**Affiliations:** 1 Faculties of Pharmacy and Medicine, Universidad San Pablo-CEU, Madrid, Spain; 2 Institute of Sport Science, University of Graz, Graz, Austria; 3 Department of Public and Occupational Health, Amsterdam Public Health Research Institute, VU University Medical Center, Amsterdam, The Netherlands; 4 Department of Gynecology and Obstetrics, Medical University Graz, Graz, Austria

## Abstract

Angiopoietin-like protein 4 (ANGPTL4) is a circulating protein involved in the regulation of adipose tissue metabolism. However, its role in obesity and pregnancy is unknown. To evaluate the relationship between gestational weight gain (GWG) and circulating concentrations of ANGPTL4 in pregnant women with overweight and obesity, weight gain and fasting maternal blood samples of thirty-one pregnant women was drawn at 15, 24 and 32 weeks of gestation. ANGPTL4 concentrations continuously rose throughout gestation, whereas VEGF and leptin did not show the same trend. NEFA and glycerol concentrations remained stable during pregnancy. In contrast, total concentrations of saturated, monounsaturated and n-6 fatty acids, but not n-3 fatty acids, increased with pregnancy. In multiple regression analysis, the increase in plasma ANGPTL4 and decrease in linoleic acid concentrations were the most significant predictors of GWG, although only ANGPTL4 was significantly associated with the weight gain from early pregnancy (area under the ROC curve was 0.80 p < 0.01(95% CI 0.61–0.99)). In conclusion, in pregnant women with overweight and obesity, an increase in plasma ANGPTL4 concentrations throughout pregnancy is positively associated with GWG and could be used as an early marker of increased susceptibility to excess gestational weight gain.

## Introduction

Throughout normal pregnancy there are major changes in maternal lipid metabolism including maternal hyperlipidemia, but how these changes affect gain of maternal fat mass and fetal growth is not completely understood^1^. The early stages of pregnancy are characterized by an anabolic condition that facilitates the accumulation of lipids in maternal depots. From studies in rats it is known that maternal hyperphagia, increased concentrations of insulin in the blood and unchanged or increased insulin sensitivity during early pregnancy result in increased synthesis of fatty acids by adipose tissue^2^. Also an increase in adipose tissue lipoprotein lipase (LPL) activity in early pregnancy^3^ enables an increased uptake of circulating triacylglycerols (TAG) by adipose tissue. Both these changes also seem to take place in human pregnancies and explain the enlargement of maternal fat depots during this stage of pregnancy^4,5^.

LPL activity is primarily governed by complex post-translational mechanisms in response to energy requirements and hormonal changes. Angiopoietin-like 4 (ANGPTL4) protein is involved in the regulation of LPL activity, preventing dimerization and subsequent activation of the enzyme^6^, although the exact mechanism is still debated. In mouse models, treatment with – or over-expression of – ANGPTL4 led to an increase in circulating TAG^7^, while in ANGPTL4 knockout mice or in those treated with anti-ANGPTL4 antibody, the concentrations of plasma TAG are low^8^. It has also been proposed that ANGPTL4 is a powerful regulator of lipid metabolism and adiposity^9^, although its role in human adipose tissue development is not well understood. Recently, we reported that in both well-controlled gestational diabetes mellitus (GDM) and healthy control women, decreased maternal ANGPTL4 concentrations is associated with increased neonatal fat mass although its concentrations in cord blood serum were independent of neonatal fat mass^10^. Human studies produced inconsistent findings regarding the notion of ANGPTL4 to act as a signal preventing fat storage, and there are also studies that failed to find an association between ANGPTL4 and TAG or non-esterified fatty acid (NEFA) concentrations^11,12^. Moreover, serum ANGPTL4 concentrations has been found higher in obese subjects without/with type 2 diabetes mellitus relative to control subjects^13^, and variants of ANGPTL4 are associated with lower plasma TAG and higher waist circumference^14^. Recently, it has been found that ANGPTL4 is upregulated in human adipocytes in response to hypoxia^15–17^, and studies using microarray-based technology have reported that the expression of ANGPTL4 is significantly upregulated in obesity, in early^18^ and late phases of adipocyte differentiation^19^, as well as in fat samples from obese subjects^20,21^.

Maternal obesity is recognized as a major risk factor for adverse pregnancy outcomes, including pre-eclampsia, gestational diabetes, gestational hypertension, cesarean delivery and large-for gestational age infants^22^. Although increased adipose tissue mass can be considered physiologically normal in pregnancy, excessive weight gain can produce a dysregulation in adipose tissue metabolism. Underlying mechanisms of excessive weight gain are not fully understood. Preventing excessive weight gain during gestation is a general goal, but especially so in women with overweight or obesity prior to pregnancy. In 2009 the Institute of Medicine (IOM) guidelines recommended a total gestational weight gain (GWG) range of 7–11 kg (15–25 lbs) for women with overweight (BMI 25–30 prior to pregnancy), and 5–9 kg (11–20 lbs) for women with obesity prior to pregnancy (BMI ≥ 30)^23^. Currently, most pregnant women with overweight and obesity exceed the IOM GWG recommendations^24^. In the last decade various programs have been launched in order to prevent excessive GWG, although they have had limited success in pregnant women with overweight and obesity prior to pregnancy^25,26^. Moreover, to date, no early metabolic markers of increased susceptibility to excessive weight gain in pregnancy have been identified.

Because of the reported association between plasma ANGPTL4 concentrations and the regulation of lipid metabolism and adiposity, we hypothesize that ANGPTL4 could be part of the mechanism for excessive gestational weight gain in overweight women. Therefore, the purpose of this study was to determine, in a population of pregnant women with overweight and obesity, the relationships between their degree of weight gain at the three trimesters of pregnancy with plasma concentration of ANGPTL4 and different lipid variables.

## Results

The characteristics of the study participants with overweight and obesity are shown in Table 1. Only 19% of the pregnant women studied showed a GWG conforming to IOM recommendations based on pre-pregnancy BMI, and more than 50% exceeded the weight that they should have gained by week 32. All neonates had appropriate weight for their gestational age, and no differences in neonatal birth weight related to maternal GWG were observed.

**Table 1:** Characteristics of participants.

	mean ± SEM	(min-max)
Age (years)	31.3 ± 0.7	(25.0–41.0)
Maternal weight pre-pregnancy (kg)	93.6 ± 2.6	(65.0–127)
Pre-pregnancy BMI (kg/m^2^)^*^	33.5 ± 0.9	(25.3–51.3)
Gestational weight gain (kg)^**^	5.33 ± 0.75	(−1.20–12,2)
Women with GWG lower than IOM recommendation	29%	
GWG in women with GWG < IOM recommendation (kg)	1.06 ± 0.60	(−1.20–2.90)
Women with GWG according IOM recommendation	19%	
GWG in women with GWG according IOM recommendation (kg)	4.16 ± 0.48	(2.90–5.50)
Women with GWG higher than IOM recommendation	52%	
GWG in women with GWG>IOM recommendation (kg)	8.31 ± 0.62	(5.70–12.2)
Gestational age at birth (weeks)	39.4 ± 0.2	(35.0–42.0)
Neonatal weight (kg)	3.43 ± 0.12	(1.62–4.92)

^*^Pre-pregnancy weight reported by pregnant women. ^**^GWG was defined as the weight gain between 15 weeks and 32 weeks. n = 31.

**Figure 1:**
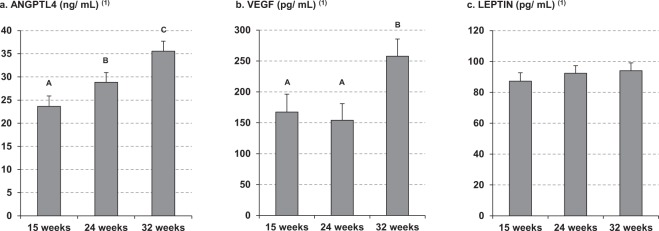
Maternal plasma concentrations of (**a**) ANGPTL4, (**b**) VEGF and (**c**) leptin at 15, 24 and 32 weeks of pregnancy, in pregnant women with overweight and obesity. Maternal parameters were adjusted by pre-pregnancy BMI. Different superscripted upper-case letters indicate significant differences (P < 0.001) between different weeks of pregnancy. All values are mean ± SEM. ^(1)^log-transformed for statistical comparisons.

The plasma ANGPTL4 concentration progressively increased throughout gestation in all women (Fig. 1a), with significant changes between all periods. However, other plasma cytokines related to vascularization and growth of adipose tissue, as vascular endothelial growth factor (VEGF) and leptin, did not show the same trend. VEGF concentration only increased at 32 weeks of gestation (Fig. 1b), whereas plasma leptin concentration did not show significant change throughout pregnancy (Fig. 1c). Plasma glucose and RBP4 concentration (an adipokine related to insulin resistance) remained stable during pregnancy, and insulin concentration and HOMA index only increased at 32 weeks of pregnancy (Supplementary Table S1). Neither non-esterified fatty acids (NEFA) nor glycerol concentrations were altered during gestation (Table 2). In contrast, as expected, plasma concentrations of TAG and phospholipids increased with gestational age (Table 2), although the phospholipids concentrations at 24 weeks did not differ from those at 32 weeks. Plasma concentration of both total saturated fatty acids (SFA) and total monounsaturated fatty acid (MUFA) also progressively increased during pregnancy (Table 2), mainly due to rising palmitic acid (PA, 16:0) and oleic acid (OA, 18:1 n-9) concentrations, respectively. Total plasma n-6 polyunsaturated fatty acids (PUFA) and their most abundant representative, linoleic fatty acid (LA, 18:2 n-6), were significantly higher at 24 than at 15 weeks of pregnancy and this difference was maintained until 32 weeks. However, the concentrations of both γ-linolenic (GLNA, 18:3 n-6) and arachidonic (AA, 20:4 n-6) acid remained virtually unchanged throughout gestation. Also the plasma concentration of total n-3 PUFA and of the n-3 fatty acids α-linolenic (ALNA, 18:3 n-3), eicosapentaenoic (EPA, 20:5 n-3) and docosahexaenoic (DHA, 22:6 n-3) acids, remained stable during pregnancy.

**Table 2:** Maternal plasma lipid concentrations at 15, 24 and 32 weeks of pregnancy, in pregnant women with overweight and obesity.

	15 weeks of pregnancy	24 weeks of pregnancy	32 weeks of pregnancy
	mean ± SEM	mean ± SEM	mean ± SEM
NEFA (mmol/L)^(1)^	0.520 ± 0.036	0.544 ± 0.033	0.555 ± 0.033
Glycerol (mmol/L)^(1)^	0.125 ± 0.010	0.135 ± 0.009	0.146 ± 0.009
TAG (mmol/L)^(1)^	1.34 ± 0.09^A^	1.62 ± 0.09^B^	2.01 ± 0.09^C^
Phospholipids (mmol/L)^(1)^	2.88 ± 0.07^A^	3.09 ± 0.06^B^	3.15 ± 0.06^B^
Total SFA (mmol/L)^(1)^	6.47 ± 0.29^A^	7.38 ± 0.27^B^	7.95 ± 0.27^C^
PA, 16:0 (mmol/L)^(1)^	4.97 ± 0.24^A^	5.72 ± 0.22^B^	6.25 ± 0.22^C^
SA, 18:0 (mmol/L)^(1)^	1.09 ± 0.04	1.17 ± 0.04	1.17 ± 0.04
Total MUFA (mmol/L)^(1)^	4.29 ± 0.22^A^	4.89 ± 0.20^B^	5.45 ± 0.21^C^
POA, 16:1 (mmol/L)^(1)^	0.469 ± 0.046^A^	0.567 ± 0.042^B^	0.636 ± 0.043^B^
OA, 18:1 n-9 (mmol/L)^(1)^	3.73 ± 0.18^A^	4.20 ± 0.17^B^	4.72 ± 0.17^C^
Total n-6 (mmol/L)^(1)^	6.33 ± 0.24^A^	6.93 ± 0.22^B^	7.05 ± 0.22^AB^
LA, 18:2 n-6 (mmol/L)^(1)^	5.09 ± 0.21^A^	5.64 ± 0.19^B^	5.87 ± 0.19^B^
GLNA, 18:3 n-6 (mmol/L)^(1)^	0.054 ± 0.006	0.062 ± 0.006	0.059 ± 0.006
AA, 20:4 n-6 (mmol/L)^(1)^	1.14 ± 0.05	1.17 ± 0.04	1.07 ± 0.04
Total n-3 (mmol/L)^(1)^	0.747 ± 0.034	0.751 ± 0.031	0.760 ± 0.031
ALNA, 18:3 n-3 (mmol/L)^(1)^	0.108 ± 0.008	0.115 ± 0.008	0.124 ± 0.008
EPA, 20:5 n-3 (mmol/L)^(1)^	0.090 ± 0.008	0.078 ± 0.008	0.079 ± 0.008
DHA, 22:6 n-3 (mmol/L)^(1)^	0.548 ± 0.024	0.547 ± 0.022	0.545 ± 0.023

Total SFA correspond to the sum of saturated 14:0, 16:0, 18:0, 20:0, 22:0 and 23:0 fatty acids. Total MUFAs correspond to the sum of monounsaturated 16:1, 18:1, 20:1 and 22:1 fatty acids. Total n-6 corresponds to the sum of 18:2 n-6, 18:3 n-6, 20:2 n-6 and 20:4 n-6 fatty acids. Total n-3 corresponds to the sum of 18:3 n-3, 20:3 n-3, 20:5 n-3 and 22:6 n-3 fatty acids. Maternal parameters were adjusted by pre-pregnancy BMI. Different superscripted upper-case letters indicate significant differences during pregnancy (p < 0.05). ^(1)^log-transformed for statistical comparisons.

**Figure 2:**
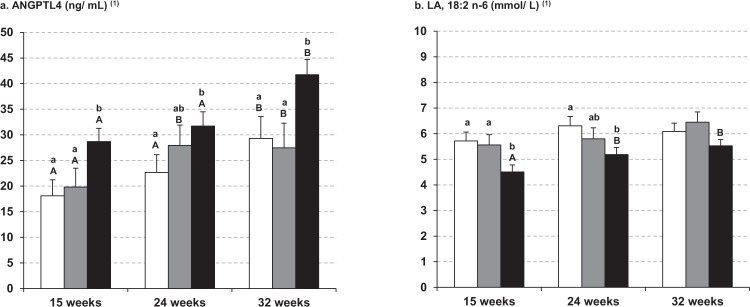
Maternal plasma concentrations of (**a**) ANGPTL4 and (**b**) linoleic acid (LA, 18:2 n-6) at 15, 24 and 32 weeks of pregnancy, in pregnant women with overweight and obesity classified by GWG conformed to the recommended rate of weight gain during such interval of pregnancy made by the IOM for their particular pre-pregnancy BMI: between 3.4 and 5.5 kg for women with overweight and between 3 and 4.6 kg for women with obesity. Open bars, data pregnant women with GWG < IOM recommendation; filled grey bars, data from pregnant women with GWG within IOM recommendation; filled black bars, data from pregnant women with GWG > IOM recommendation. For each subgroup, different superscripted upper-case letters indicate significant differences (P < 0.001) between different weeks of pregnancy, whereas different superscripted lower-case letters indicate significant differences (P < 0.001) between subgroups at each stage of gestation analyzed (i.e. 15, 24 and 32 weeks of pregnancy). Maternal parameters were adjusted by pre-pregnancy BMI. All values are mean ± SEM. ^(1)^log-transformed for statistical comparisons.

**Figure 3:**
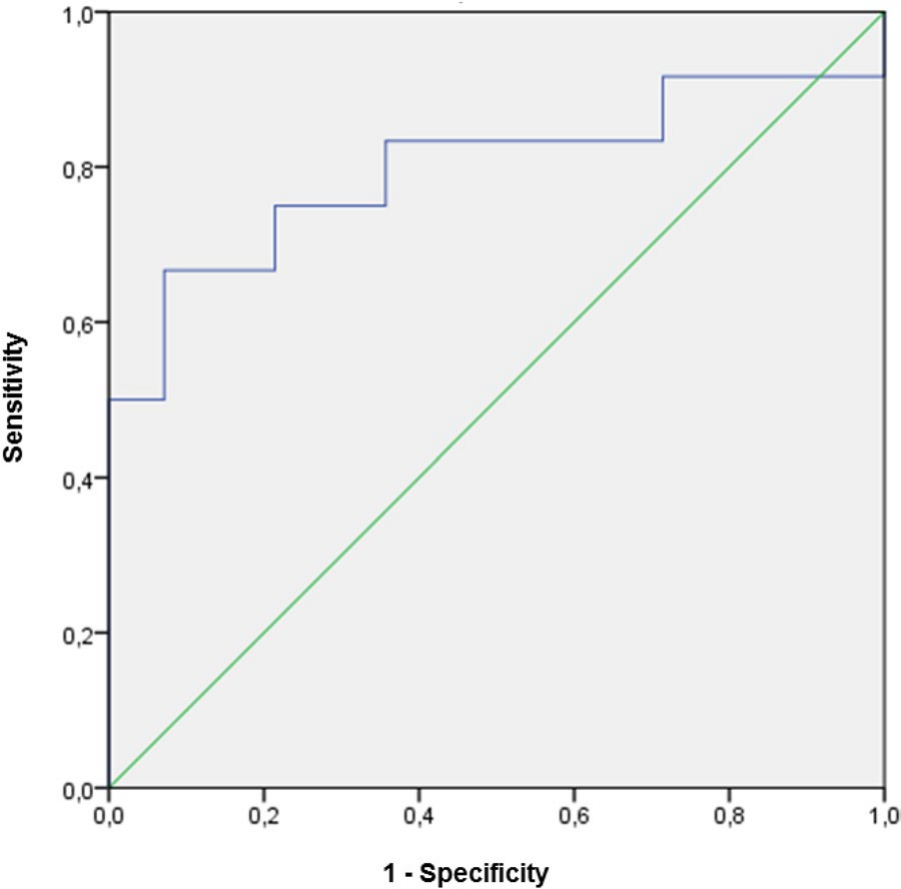
ROC curve of ANGPTL4 concentration at week 15 of pregnancy for predicting gestational weight gain higher than recommended. Standard error = 0.01; 95% CI = 0.61 to 0.99; P < 0.01.

When the individual plasma concentration of these variables were correlated with GWG throughout pregnancy, only ANGPTL4 was significantly and positively associated with GWG at the three time periods of pregnancy (Table 3). At 32 weeks of pregnancy, leptin, stearic (SA,18:0), palmitoleic (POA,16:1 n-7), GLNA (18:3 n-6) and ALNA (18:3 n-3) acid concentrations were positively correlated with GWG, whereas LA (18:2 n-6) was the only variable negatively correlated with GWG in this period. ANGPTL4 was positively correlated with leptin concentration, but contrary to expectations, no association was found between ANGPTL4 and TAG or NEFA in any period studied (data not shown). Moreover, when associations of GWG with all the variables were analyzed by multiple regression, more than 67% of the variation in GWG was significantly and positively associated with the increase in both ANGPTL4 and SA (18:0), and negatively associated with the LA (18:2, n-6) concentrations (Table 4) without significant association with any of the other variables studied. Thus, women with highest GWG had higher plasma concentrations of ANGPTL4 than those with low or normal GWG at the three time periods of pregnancy (Fig. 2a). In contrast, LA (18:2, n-6), was lower in maternal plasma of pregnant women with the highest GWG versus the two other subgroups, although it only reached statistical significance at 15 and 24 weeks (Fig. 2b). Curiously, pregnant women who showed disturbances in GWG (those with low and highest GWG), at early pregnancy had significant lower concentration of glucose, insulin and RBP4, as well as HOMA-IR values than those with the recommended GWG (Supplementary Fig. S1), but these differences disappeared as pregnancy progressed. Only insulin (Supplementary Fig. S1B) and HOMA-IR (Supplementary Fig. S1C) were significantly higher at 32 weeks than those found at earlier times of pregnancy, in pregnant women whose GWG was higher than recommended. Finally, the ability of ANGPTL4, stearic and linoleic acid to predict excessive weight gain was assessed by receiver operating characteristic (ROC) analysis. Plasma ANGPTL4 concentration at week 15 was identified as the only predictor of highest GWG (Fig. 3). This was reflected by the area under the ROC curve (AUC) of 0.80 (standard error = 0.10; 95% CI = 0.61 to 0.99; P < 0.01), indicating a satisfactory discriminatory capacity for ANGPTL4.

**Table 3:** Correlation coefficient (r) between gestational weight gain at 15, 24 and 32 weeks of pregnancy and metabolic parameters in maternal plasma of pregnant women with overweight and obesity.

	Weight gain at 15 weeks of pregnancy	Weight gain at 24 weeks of pregnancy	Weight gain at 32 weeks of pregnancy
ANGPTL4 (ng/mL)^1^	0.441 (0.028)	0.395 (0.042)	0.443 (0.039)
Leptin (pg/mL)^1^	0.022	0.190	0.676 (0.001)
VEGF (pg/mL)^1^	−0.221	−0.003	−0.197
NEFA (mmol/L)^1^	−0.166	0.161	0.015
Glucose (mmol/L)	0.092	−0.150	−0.125
RBP4 (μg/mL)	0.355	0.020	0.053
Insulin (pmol/L)^1^	0.136	0.166	0.085
HOMA^1^	0.147	0.115	0.055
Glycerol (mmol/L)^1^	0.114	0.216	0.034
TAG (mmol/L)^1^	−0.012	0.255	−0.075
Phospholipids (mmol/L)^1^	0.174	0.304	0.280
PA, 16:0 (mmol/L)^1^	0.150	0.284	0.242
SA, 18:0 (mmol/L)^1^	0.346	0.197	0.434 (0.034)
POA, 16:1 (mmol/L)^1^	0.128	0.256	0.429 (0.036)
OA, 18:1 n-9 (mmol/L)^1^	0.142	0.235	0.311
LA, 18:2 n-6 (mmol/L)^1^	0.028	−0.148	−0.489 (0.015)
GLNA, 18:3 n-6 (mmol/L)^1^	−0.141	0.310	0.421 (0.040)
AA, 20:4 n-6 (mmol/L)^1^	0.282	0.165	0.311
ALNA, 18:3 n-3 (mmol/L)^1^	−0.141	0.310	0.421 (0.040)
EPA, 20:5 n-3 (mmol/L)^1^	0.329	0.243	0.168
DHA, 22:6 n-3 (mmol/L)^1^	0.168	0.118	0.125

In parenthesis, p value for significant correlation (p < 0.05). ^1^log transformed for statistical comparisons. Weight gain at 15 weeks is the difference between measured weight at 15 weeks of pregnancy and pre-pregnancy self-reported weight. Weight gain at 24 weeks is the difference between 24 and 15 weeks of pregnancy. Weight gain at 30 weeks is the difference between 30 and 24 weeks of pregnancy.

**Table 4:** Multiple Regression Analysis of Maternal Contributors to Gestational Weight Gain (GWG).

Selected predictors	β	P	R^2^ model
ANGPTL4^1^	4.53	0.01	67.3
Stearic acid (SA, 18:0)^1^	14.2	0.0002	
Linoleic acid (LA, 18:2, n-6)^1^	−5.73	0.03	

GWG was defined as the weight gain between 15 weeks and 32 weeks.

The model included the following independent variables: BMI pre-pregnancy, ANGPTL4^1^, leptin^1^, VEGF^1^, triacylglycerol^1^, NEFA^1^, glycerol^1^, 16:0^1^, 16:1 n-7^1^, C18:0^1^, 18:1 n-9^1^, 18:3 n-3^1^, 20:5 n-3^1^, 22:6 n-3^1^, 18:2 n-6^1^, 18:3 n-6^1^ and 20:4 n-6^1^. In the backward selection, variables were removed for F values lower than 4. Only statistically significant predictors are shown. ^1^log transformed skewed data.

## Discussion

Obesity among pregnant women has increased alarmingly in recent years, as has the prevalence of obesity in the general population, and most pregnant women with overweight and obesity in developed countries exceed the IOM recommendations for GWG^26^. Therefore, an early metabolic marker for increased susceptibility to GWG is highly desirable. This would allow for targeted intervention for the prevention of excessive GWG. Our study shows for the first time that plasma ANGPTL4 concentrations measured in early pregnancy may serve as such a marker in pregnant women with overweight and obesity. Indeed, higher ANGPTL4 concentrations were found as early as 15 weeks of pregnancy in those pregnant women with overweight and obesity that had the highest GWG compared with those with low or appropriate GWG. This result appears against the known role of ANGPTL4 decreasing adipose tissue LPL activity^27^, but it may be a consequence of the dysregulation of adipose tissue in pregnant women with overweight and obesity, having excessive GWG mediated by the effect of ANGPTL4. Since neither the plasma concentrations of TAG or phospholipids were related with GWG, ANGPTL4 nor leptin, a mechanistic contribution of ANGPTL4 to adipose tissue accretion mediated by LPL in these women appears unlikely. Under non-pregnant conditions such as fasting^28^, exercise^29^ and diabetic insulin-resistance^10,30^, ANGPTL4 has been related to their active adipose tissue lipolysis. However, in agreement with previous studies^11^, we did not find here any relationship between the plasma concentration of NEFAs with ANGPTL4. In addition, plasma concentrations of NEFAs and glycerol were independent of GWG, not supporting the contention that alterations in lipolysis caused by the changes in ANGPTL4 concentration, could be responsible for the differences observed in GWG.

The results of this study suggest that excessive, i.e. higher than recommended by IOM, weight gain during pregnancy is related to disturbances in maternal adipose tissue. In women with overweight and obesity in pre-pregnancy, the rate at which maternal weight and fat mass increase during gestation is normally lower than in women without obesity^31^. As is the case in normal pregnancy, GWG in pregnant women with overweight and obesity is known to correlate with maternal fat deposition^32^. In our study, the concentration of leptin, a marker of adipose tissue accretion in both pregnant and nonpregnant conditions^33–35^ did not change significantly throughout pregnancy, in keeping with the lower tendency for fat mass accumulation of these pregnant women. However, at 32 weeks of gestation leptin concentration was significantly correlated with GWG, indicating that some of the GWG at late pregnancy corresponds to adipose tissue accretion. We found here that it is in pregnant women with GWG higher than recommended where ANGPTL4 concentration was higher already at week 15 of pregnancy. Different from its role in lipid and adipose tissue metabolism, ANGPTL4 can also act as an early pro-angiogenic cytokine inducing adipocyte differentiation and endothelial cell growth necessary for adipose tissue expansion^20,36^. The effect of ANGPTL4 on adipose tissue accretion would later be reinforced by the synthesis of other cytokines such as VEGF, whose concentration we found to increase in the last trimester of pregnancy. These results are in accordance with other studies reporting that ANGPTL4 expression is significantly upregulated in obesity, in the early^18^ and the later phases of adipocyte differentiation^19,37^, and also in adipose tissue cells from subjects with obesity^21,36^.

In addition to the changes in the plasma concentration of ANGPTL4, we found that GWG in pregnant women with obesity was related negatively to LA (18:2, n-6) and positively to saturated fatty acids, especially SA (18:0), concentrations in plasma. No differences were observed in the plasma concentrations of n-3 fatty acids. Although these results are consistent with other studies that found low plasma LA, but higher concentrations of other n-6 PUFAs, in adults with overweight and obesity (reviewed in^38^), only few studies have examined the relationship between the fatty acid profile and obesity during pregnancy. In accordance with our results, lower plasma LA but higher arachidonic and palmitic acid concentrations at 20 weeks of pregnancy were previously found in pregnant women with obesity with higher GWG^39^. However, the mechanistic link between these changes in fatty acid profile and obesity is unclear.

In conclusion, our results support the possibility that plasma ANGPTL4 concentrations could be used as an early indicator of future excessive weight gain during pregnancy. We propose that an early increase in ANGPTL4 production in response to a decrease in linoleic acid concentration and an increased stearic acid concentration could induce angiogenesis and adipogenesis in pregnant women with overweight and obesity, making them more susceptible to excessive GWG during pregnancy.

## Research Design and Methods

### Study subjects

The sample studied consisted of 31 pregnant women with overweight (pre-pregnancy 25 ≤ BMI < 30) and obesity (pre-pregnancy BMI ≥ 30), who were at increased risk of GDM. The women were recruited in midwife practices and hospitals in Amsterdam, the Netherlands, between January 2007 and January 2011, as has been described in detail previously^40^. Women were considered to be at increased risk of GDM if they had overweight or obesity and had at least one of the following characteristics: (1) history of macrosomia (offspring with a birth weight above the 97^th^ percentile for a given gestational age); (2) history of GDM; or (3) a first-degree relative with type 2 diabetes mellitus. Exclusion criteria included recruitment after 20 weeks of gestation; under 18 years or age; inadequate knowledge of the Dutch language; diagnosis of GDM at baseline; hypertension; pulmonary, cardiac, hepatic or renal impairment; alcohol or drugs abuse and use of medication that affects insulin secretion or insulin sensitivity. For the purpose of this study women with GDM diagnosed during the course of pregnancy were also excluded. All participating mothers gave informed written consent after having received verbal and written information on the study. The study protocol was approved by the Medical Ethics Committee of VU University Center in Amsterdam (2007/133).

Pre-pregnancy weight was self-reported, but maternal body weight throughout gestation was measured by calibrated electronic scales while participants were only wearing indoor clothing and no shoes. Maternal height was measured (in bare feet) by a wall-mounted height scale to calculate the body mass index.

Gestational weight gain was defined as the weight gained between the measurements at 15 and 32 weeks. Conforming with the IOM recommendations^23^, an adequate gestational weight during this interval was between 3.4 and 5.5 kg for overweight women and between 3 and 4.6 kg for women with obesity.

### Blood samples

Maternal blood samples were drawn in heparinized tubes from the antecubital vein after the participant had fasted for at least 10 h. Maternal blood samples were centrifuged (1500 × g at 4C for 25 min) and aliquots of plasma were immediately stored at −80C until analysis. None of the samples used in the study showed hemolysis.

### Analytical determinations

Plasma ANGPTL4 (USCN Life Science, Wuhan, China; intra-assay variations of <10% and inter-assay variation of <12%), leptin (eBioscience, San Diego CA, USA; intra-assay variation 5.7% and inter-assay variation 6.9%), retinol binding protein-4 (RBP4) (AdipoGen, Seoul, Korea; intra-assay variation 3.4% and inter-assay variation 7.1%), and VEGF (IBL-Clontech, Gunma, Japan; intra-assay variation 5.9% and inter-assay variation 9.4%) were determined using sandwich ELISA kits. Plasma insulin was measured by an immunometric assay (Luminescence, Advia Centaur; Siemens Medical Solutions Diagnostics, Deesfield, Illinois); insulin sensitivity was calculated from the homeostasis model assessment (HOMA). Plasma glucose, TAG (Roche Diagnostics GmbH, Germany), NEFA (Wako Chemicals GmbH, Neuss, Germany) and glycerol (Sigma, St. Louis, MO) were determined enzymatically using commercial kits.

For the analysis of fatty acid profiles, plasma lipids were extracted in chloroform/methanol (2:1 by volume) containing 0.005 % BHT and an internal standard of nonodecenoic acid (19:1). Dried lipid extracts were subjected to methanolysis for 2.5 h at 80 °C in methanol:toluene (4:1 by volume) containing acetyl chloride and the methyl esters were analyzed on a Perkin Elmer gas chromatograph (Autosystem; Norwalk, CT) in the presence of methyl-heptadecanoate (17:0) as a reference standard, as previously reported^41^.

All methods were performed in accordance with the relevant guidelines and regulations.

### Statistics

Results are expressed as means ± SEM. Statistical difference between subgroups was determined by ANOVA, after adjustment for pre-gestational BMI as a possible confounding factor; when differences were statistically significant, multiple comparisons were performed using the Tukey post hoc test. Given their skewed distributions, concentrations of ANGPTL4, VEGF, leptin, TAG, NEFA and glycerol were log-transformed before statistical comparison. Correlations were tested using Pearson’s method with the log-transformed data. To ascertain the independent predictors of gestational weight gain, stepwise multiple regressions with backward selection were performed. To determine the association between the early ANGPTL4 concentration and gestational weight gain higher than recommended, a receiver operating characteristic (ROC) curve was generated. All statistical analyses were performed using a computer software package (SPSS Statistics 24, Chicago, IL, USA). The statistics at p-value < 0.05 was considered significant.

## Electronic supplementary material

Supplementary information
