# Supplementary material for: Angiopoietin-like protein 4 (ANGPTL4) is related to gestational weight gain in pregnant women with obesity
Source: Sci Rep. 2018 Aug 20;8:12428. doi: 10.1038/s41598-018-29731-w (PMC6102233; doi:10.1038/s41598-018-29731-w)
Supplement: Supplementary file 1 — Supplementary information [file 41598_2018_29731_MOESM1_ESM.pdf]

Supplementary information

Angiopoietin-like protein 4 (ANGPTL4) is related to gestational weight gain in pregnant women with obesity

Henar Ortega-Senovilla, Mireille N.M. van Poppel, Gernot Desoye, Emilio Herrera

Supplementary *Table S1.* *Maternal plasma glucose, RBP4, insulin concentrations and HOMA-IR at 15, 24 and 32 weeks of pregnancy, in pregnant women with overweight and obesity.*

	15 weeks of pregnancy<br>mean±SEM	24 weeks of pregnancy<br>mean±SEM	32 weeks of pregnancy<br>mean±SEM
Glucose (mmol/ L)	4.70±0.09	4.68±0.08	4.71±0.08
RBP4 (µg/ mL)	73.8±3.6	71.0±3.2	67.1±3.3
Insulin (pmol/ L) [(1)]	72.6±6.8 [A]	83.0±7.0 [A]	99.0±8.2 [B]
HOMA-IR [(1)]	2.62±0.28 [A]	2.96±0.24 [A]	3.74±0.24 [B]

Maternal parameters were adjusted by pre-pregnancy BMI. Different superscripted upper-case letters indicate significant differences during pregnancy ($p<0.05$). [(1)] log-transformed for statistical comparisons.

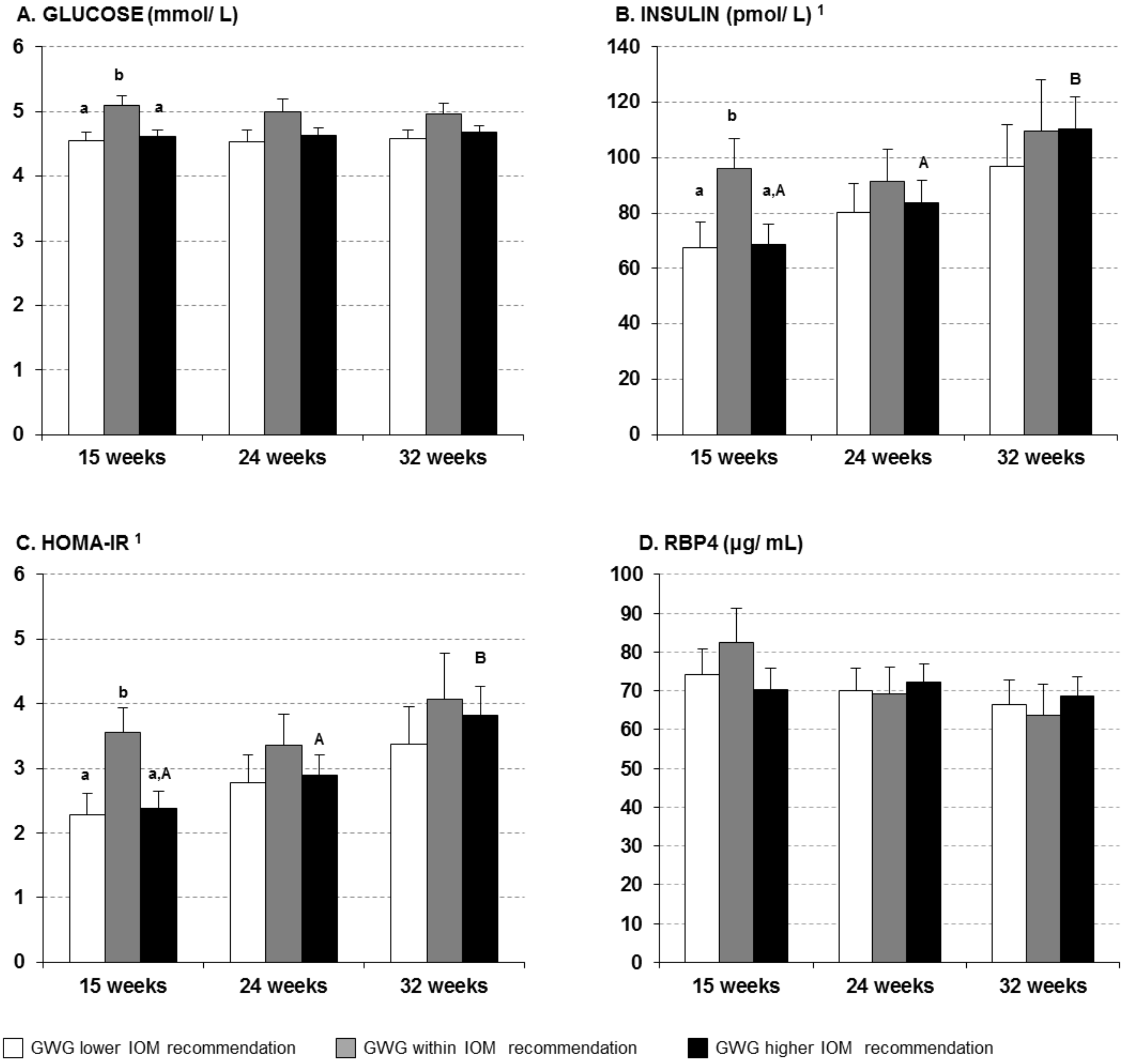

Supplementary Figure S1. Maternal plasma concentrations of (A) Glucose, (B) Insulin, (C) HOMA-IR and (D) RBP-4 at 15, 24 and 32 weeks of pregnancy, in pregnant women with overweight and obesity classified by GWG conformed to the recommended rate of weight gain during such interval of pregnancy, made by the IOM for their particular pre-pregnancy BMI: between 3.4 and 5.5 kg for women with overweight and between 3 and 4.6 kg for women with obesity. Open bars, data pregnant women with GWG < IOM recommendation; filled grey bars, data from pregnant women with GWG within IOM recommendation; filled black bars, data from pregnant women with GWG > IOM recommendation. For each subgroup, different superscripted upper-case letters indicate significant differences (P<0.001) between different weeks of pregnancy, whereas different superscripted lower-case letters indicate significant differences (P<0.001) between subgroups at each stage of gestation analyzed (i.e. 15, 24 and 32 weeks of pregnancy). Maternal parameters were adjusted by pre-pregnancy BMI. All values are mean ± SEM. (1) log-transformed for statistical comparisons.
